# Genomics Insights into *Pseudomonas* sp. CG01: An Antarctic Cadmium-Resistant Strain Capable of Biosynthesizing CdS Nanoparticles Using Methionine as S-Source

**DOI:** 10.3390/genes12020187

**Published:** 2021-01-27

**Authors:** Carla Gallardo-Benavente, Jessica L. Campo-Giraldo, Juan Castro-Severyn, Andrés Quiroz, José M. Pérez-Donoso

**Affiliations:** 1 Programa de Doctorado en Ciencias de Recursos Naturales, Universidad de La Frontera, 4780000 Temuco, Chile; carlagallardo6@gmail.com; 2 Centro de Excelencia en Investigación Biotecnológica Aplicada al Medio Ambiente (CIBAMA), Facultad de Ingeniería y Ciencias, Universidad de La Frontera, 4780000 Temuco, Chile; 3 BioNanotechnology and Microbiology Lab, Center for Bioinformatics and Integrative Biology, Facultad de Ciencias de la Vida, Universidad Andres Bello, 8320000 Santiago, Chile; jek2908@gmail.com; 4 Laboratorio de Microbiología Aplicada y Extremófilos, Facultad de Ingeniería y Ciencias Geológicas, Universidad Católica del Norte, 1240000 Antofagasta, Chile; jsevereyn@gmail.com; 5 Departamento de Ciencias Químicas y Recursos Naturales, Facultad de Ingeniería y Ciencias, Universidad de La Frontera, 4780000 Temuco, Chile

**Keywords:** Antarctic bacteria, nanoparticle biosynthesis, comparative genomics, volatile sulfur compounds

## Abstract

Here, we present the draft genome sequence of *Pseudomonas* sp. GC01, a cadmium-resistant Antarctic bacterium capable of biosynthesizing CdS fluorescent nanoparticles (quantum dots, QDs) employing a unique mechanism involving the production of methanethiol (MeSH) from methionine (Met). To explore the molecular/metabolic components involved in QDs biosynthesis, we conducted a comparative genomic analysis, searching for the genes related to cadmium resistance and sulfur metabolic pathways. The genome of *Pseudomonas* sp. GC01 has a 4,706,645 bp size with a 58.61% G+C content. *Pseudomonas* sp. GC01 possesses five genes related to cadmium transport/resistance, with three P-type ATPases (*cadA*, *zntA*, and *pbrA*) involved in Cd-secretion that could contribute to the extracellular biosynthesis of CdS QDs. Furthermore, it exhibits genes involved in sulfate assimilation, cysteine/methionine synthesis, and volatile sulfur compounds catabolic pathways. Regarding MeSH production from Met, *Pseudomonas* sp. GC01 lacks the genes *E4.4.1.11* and *megL* for MeSH generation. Interestingly, despite the absence of these genes, *Pseudomonas* sp. GC01 produces high levels of MeSH. This is probably associated with the *metC* gene that also produces MeSH from Met in bacteria. This work is the first report of the potential genes involved in Cd resistance, sulfur metabolism, and the process of MeSH-dependent CdS QDs bioproduction in *Pseudomonas* spp. strains.

## 1. Introduction

Antarctica is one of the most extreme ecosystems for the development of life on earth [1]. The harsh conditions that characterize this environment include low temperatures, high dehydration rates, high radiation, and low nutrient availability [2]. However, many microorganisms have adapted to colonize this environment by developing unique strategies to survive under these conditions. Because of this, the Antarctic continent is of great interest as a source of biodiversity and, therefore, of new biotechnological and bioactive compounds, such as enzymes, proteins, and secondary metabolites [3,4,5]. In this context, the use of Antarctic microorganisms as bio-factories for the production of metal-based nanocrystals or quantum dots (QDs) has been explored during the last years [6,7,8]. 

QDs are fluorescent semiconductor nanoparticles generally composed of CdS, CdSe, ZnS, ZnTe, CdTe, InP, CuInS2, or GaAs, with sizes below 20 nm [9,10,11,12]. These nanoparticles exhibit outstanding characteristics, including broad absorption, size-dependent emission color, narrow emission profile, resistance to photobleaching, strong luminescence, and long luminescent lifetimes [12,13,14]. The remarkable properties of QDs are associated with the nanocrystals’ size and composition [11,12]. They can be effectively tapped for several applications, such as for imaging techniques [15], solar cells [16,17], optoelectronics [18], and quantification of different molecules [19,20], among others.

The biosynthesis or biological production of QDs using microorganisms has emerged as an eco-friendly, cost effective, and highly biocompatible alternative [21,22]. Bacterial biosynthesis of cadmium-based QDs has been extensively studied during the last years because of their simplicity and the distinctive optical properties of the nanoparticles produced [7,23,24,25]. Most biosynthesis methods described to date have been associated with sulfur-containing molecules, such as peptides, antioxidant thiols, and hydrogen sulfide (H_2_S). In general, H_2_S has been reported as a source of sulfide anion (S^2−^) that interacts with the cadmium ion (Cd^2+^) for the formation of CdS QDs [21,24,26,27]. Nevertheless, despite the knowledge generated during the last years, the mechanism involved in CdS nanoparticle biosynthesis is still unclear.

*Pseudomonas* is a genus of bacteria known for its metabolic versatility and capacity to inhabit many environments, including Antarctica [28,29,30]. Recent research has reported that Antarctic bacteria from the *Pseudomonas* genus can biosynthesize CdS nanoparticles at low temperatures (15 °C) [6]. *Pseudomonas* sp. GC01, previously reported as *Pseudomonas fragi* GC01 [6], is a psychrotolerant bacterial strain isolated from Deception Island (South Shetland archipelago, Antarctica), highly resistant to cadmium (minimal inhibitory concentration (MIC) of 1.8 mM CdCl_2_), and with the ability to biosynthesize CdS QDs [6,8]. *Pseudomonas* sp. GC01 strain can biosynthesize QDs inside cells in the presence of sulfate, sulfite, thiosulfate, cysteine (Cys), and methionine (Met) as the sole sulfur sources. On the other hand, extracellular biosynthesis of CdS QDs only occurs in the presence of Cys and Met. Interestingly, this bacterium biosynthesizes CdS QDs through a novel mechanism that uses methanethiol (MeSH) instead of H_2_S as a sulfur source for nanocrystal formation [8]. In this work, we studied the genome of *Pseudomonas* sp. GC01 to understand the molecular and metabolic components involved in their unique mechanism to biosynthesize CdS QDs through a comparative genomic analysis.

## 2. Materials and Methods

### 2.1. Bacterial Isolation and Growth Conditions

The *Pseudomonas* sp. GC01 strain used in this work was isolated from a soil sample obtained from Deception Island in the South Shetland archipelago, Antarctica (S62°58′06.2″ W 60°42′32.5″), during the 48th Chilean Antarctic Expedition (ECA) organized by the Chilean Antarctic Institute (INACH), and previously identified as *Pseudomonas fragi* GC01 [6]. Bacterial cultures were grown in Luria Bertani (LB) medium [31] at 15 °C. 

### 2.2. DNA Extraction, Sequencing, and Assembly

DNA was isolated with the Genomic DNA kit (UltraClean Microbial DNA Isolation Kit, Mo Bio Laboratories, Inc, Carlsbad, CA, USA) according to the manufacturer’s instructions. Following this, genomic libraries were constructed using the NanoTru-Seq DNA kit (for a pair-ended with an insert size average of 420 pb). Next, 1.6 pM of the libraries were loaded, and the run was performed in a MiSeq platform (Illumina). The resulting reads were filtered and trimmed by using Trimmomatic v0.30 [32], with filters of quality (Q < 30), length (<100), ambiguities (0Ns), and adapters that were cut. Moreover, the filtered reads were de-novo assembled using the SPAdes v3.7 software [33]. Hence, assembly quality and completeness/contamination were evaluated using Quast v5.0.2 [33] and CheckM v1.1.2 [34] software, respectively. The complete genome sequence of *Pseudomonas* sp. strain GC01 has been deposited in GenBank under the accession number JABEMH000000000.1 (BioProject: PRJNA629082).

### 2.3. Genome Functional Description

Functional assignations of the assembled genome were made through annotation with Prokka v1.13.3 [35] and EggNOG Mapper v2.0.1 [36] software. EggNOG orthologues prediction was inferred with the diamond mapping strategy and the orthologues selected was restricted to one-to-one annotation. The chromosome topology was drawn using DNAPlotter v18.0.0 [37]. Moreover, the clusters of orthologous groups (COG) classification of the *Pseudomonas* sp. CG01 predicted proteins were visualized through the ggplot2 R package [38].

### 2.4. Pseudomonas Genomic Dataset

A total of 28 *Pseudomonas* strains (including *Pseudomonas* sp. CG01) were used for comparative analyses. The other 27 genomes were extracted from GenBank (Supplementary Table S1), selected trying to capture considerable diversity, cold environment origin, and the presence of interesting phenotypic capacities (such as the production of volatile sulfur compounds). All 28 genomes were re-annotated with Prokka v1.13.3 [35] and EggNOG mapper v2.0.1 [36] to have a comparable set.

### 2.5. Genetic Relationships and Pan-Genome Analysis

For whole-genome comparisons, the average nucleotide identity (ANI) was calculated for the dataset, in an all-against-all pairwise manner, using pyani (Python3 module [39]), with a BLASTn approach [40] (Altschul et al., 1990). The results were visualized using pheatmap v1.0.12 R packages [41]. Moreover, the pan genome was calculated, defining the compartments by clustering the proteins families into ortholog groups based on their sequence similarity using GET_HOMOLOGUES [42] with the orthoMCL v1.4 [43] algorithm. The core genome was composed of the protein clusters present in ≥26 (of the 28) genomes. The accessory genome was composed of those protein clusters present in ≤3 genomes, and the clusters present in between 4 and 25 genomes were classified as the disposable genome. Moreover, starting with the alignment of the core-genome clusters, a phylogenetic reconstruction was calculated using the PARS program from the PHYLIP v3.6 package to produce a parsimony tree, which was visualized using FigTree v1.4.3.

### 2.6. Phenotype Gene Search 

To identify the metal resistance/tolerance genes (especially bivalent cations) and their distribution among the genomes set, a BLASTp approach was used; also, the BacMet: Metal Resistance Experimental Database v2.0 [44] was targeted with the 28 *Pseudomonas* genomes, considering the e-value (<1E^−03^), query coverage (>75%), and identity (>70%) filters. Besides, a second database was constructed for this research based on evidence in the literature (Supplementary Table S2). On the other hand, the genes related to the interest pathways were extracted from each genome using the KEGG identifiers (from the EggNOG annotations). Then, sulfur metabolism (map00920) and cysteine/methionine metabolism (ko00270) were converted to KEGG molecular networks using KEGG Mapper [45]. Finally, the results were visualized using the ggplot2 R package [38].

## 3. Results and Discussion

### 3.1. Genomic Features of Antarctic Pseudomonas sp. GC01

**Figure 1:**
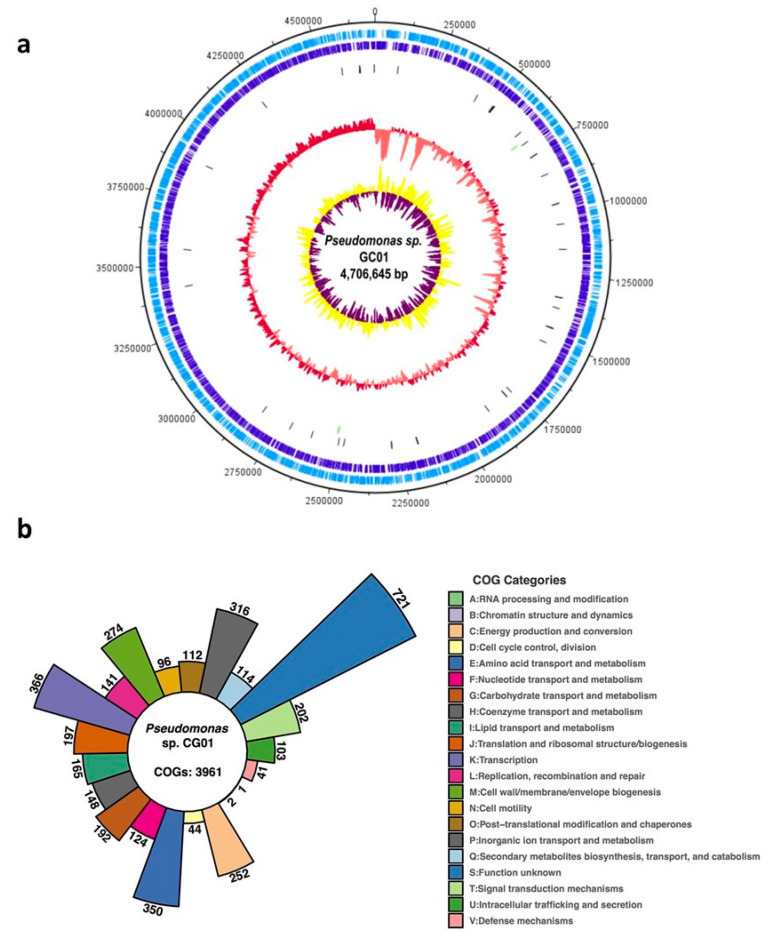
Genome characteristics of *Pseudomonas* sp. GC01. (**a**) Complete chromosome map of *Pseudomonas* sp. GC01. The chromosome map comprises six circles. The dark-blue and light-blue circles show the positions of the protein-coding genes on the plus and minus strands. The black bars on the third circle represent tRNA genes. The green bars on the fourth circle represent rRNA genes. The pink/red circle shows the GC content. The purple/yellow circle shows the GC skew. (**b**) Distribution of COG categories on the *Pseudomonas* sp. GC01 predicted proteins. The figure shows the number of CDSs assigned in each COG category depicted by color.

The draft genome sequence of *Pseudomonas* sp. GC01 was obtained by Illumina sequencing, and the assembly was deposited in GenBank (accession number JABEMH000000000.1). The size of the *Pseudomonas* sp. GC01 genome was 4,706,645 bp with a guanine-cytosine (GC) content of 58.6%. The genome annotation yielded 4875 predicted coding sequences (CDSs, including 2411 hypothetical proteins), 49 transference RNA (tRNA), and three ribosomal RNA (rRNA) genes on 2004 contigs (N50: 3572 bp; Figure 1a). The functional classification of the CDSs was performed based on clusters of orthologous groups of proteins (COGs) [46]. From the total number of CDSs (4875) found in *Pseudomonas* sp. GC01, 3961 (81.3%) of them were classified in COGs functional categories, of which 721 were functionally unknown (COG S) (Figure 1b), leaving 914 that could not be classified. The largest COG categories were transcription (COG K) with 366 CDSs, corresponding to 9.2% of the total, followed by amino acid transport and metabolism (COG E), inorganic ion transport (COG P) and metabolism, and cell wall/membrane/envelope biogenesis (COG M), with 8.8% (350 CDSs), 7.9% (316 CDSs), and 6.9% (274 CDSs), respectively (Figure 1b).

### 3.2. Genetic Relationships and Pan-Genome Analysis

Comparative genome analysis of *Pseudomonas* sp. GC01 was performed using 27 *Pseudomonas* genome sequences available in GenBank (Supplementary Table S1). The selection criteria of the *Pseudomonas* strains were mainly cold environment origin and Blast sequence similarity with *Pseudomonas* sp. GC01. *Pseudomonas* strains with interesting phenotypic characteristics, such as VSC production (*P. deceptionensis* species; [8,47]), and the reference strain *P. aeruginosa* PAO1 [48,49] were also included in the study. 

**Figure 2:**
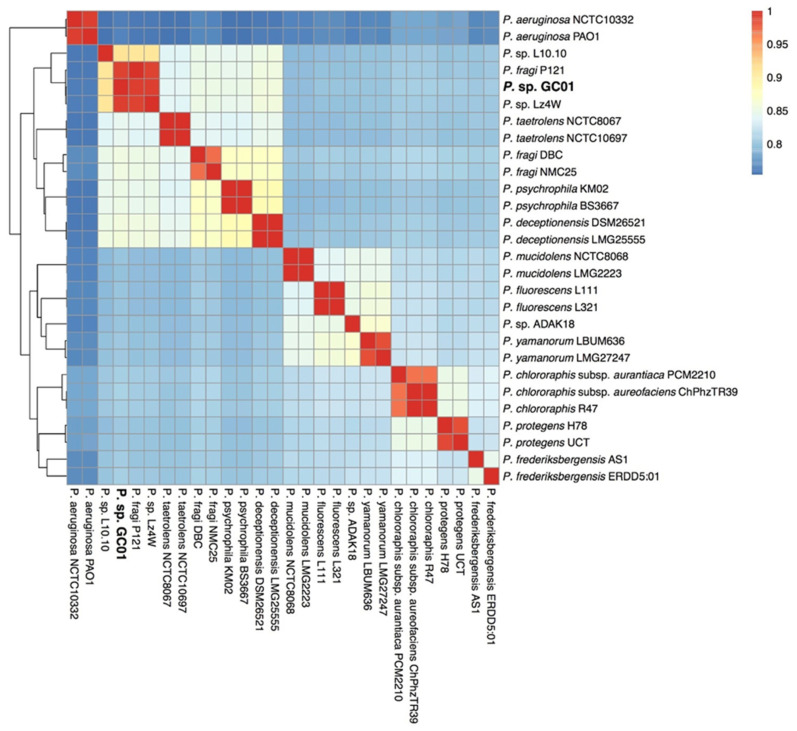
Heatmap displaying the relationships (hierarchical clustering) between the 28 *Pseudomonas* strains based on ANI analysis. The color gradients show the percentage of identity shared by each pair of genomes, from lowest (blue) to highest (red).

Genome similarity of the 28 *Pseudomonas* strains studied was determined by average nucleotide identity (ANI) analysis [39]. The ANI values ranged from 75.3% to 99.9%, indicating high diversity between the entire genomes set evaluated (Figure 2). A 99% sequence identity was determined between *Pseudomonas* sp. GC01, *Pseudomonas* sp. Lz4W [50], and *P. fragi* P121 [51], suggesting that these strains belong to the same species. However, it is interesting that the *P. fragi* P121 strain did not group with the other two *P. fragi* strains included in the dataset, only sharing 85% identity with both strains. Moreover, the *Pseudomonas* sp. GC01 also closely resembles *Pseudomonas* sp. L.10.10 [52], with a 91% sequence identity, followed by an 85% sequence identity to *P. fragi* DBC [53] and two strains of *P. deceptionensis* LMG25555 and DSM26521 [54,55]. Interestingly, the strains with the higher similarity to *Pseudomonas* sp. GC01 were isolated from cold environments, such as the Arctic (*P. fragi* P121 [51]) and Antarctica (*Pseudomonas* sp. Lz4W, *Pseudomonas* sp. L.10.10, and *P. deceptionensis* LMG25555; [50,52,54]. The *P. aeruginosa* strains were also the most divergent and ancestral branch among the set.

**Figure 3:**
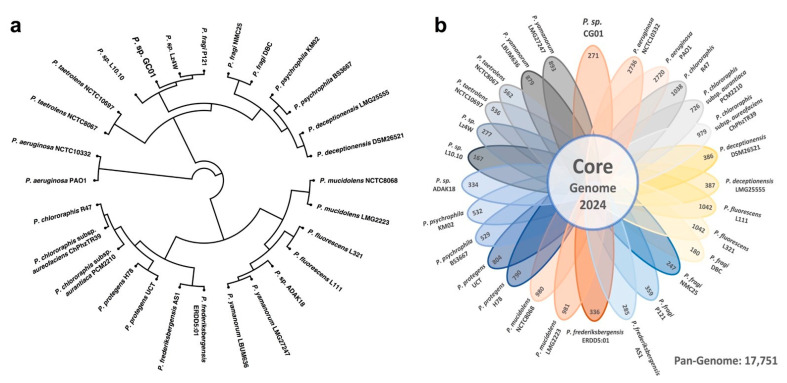
Phylogeny and pan-genome of 28 *Pseudomonas* strains. (**a**) Circular phylogenetic tree showing the relationships between all *Pseudomonas* strains, inferred based on the core-genome sequences alignment. (**b**) Flower diagram representing the amount of core and accessory clusters for each *Pseudomonas* strain considered in the pan-genome.

The phylogenomic results further confirm the segregation of the *Pseudomonas* strains in several divergent modules (Figure 3a), where the *P. aeruginosa* strains are the most distant and ancient of the set. The *Pseudomonas* sp. GC01 strain shares a clade with the *P. fragi* P121, *P.* sp. Lz4W, and *P.* sp. L.10.10 strains, inside a branch mostly composed of strains from cold environments [50,51,52]. A pan-genome analysis was performed to determine if the phenotypic differences could arise from the genotypic diversity between the *Pseudomonas* set. The pan-genome was composed of 17,751 clusters, with only 2024 clusters (11.4%) belonging to the core-genome compartment (Figure 3b), a result that supports the high diversity previously established between these 28 genomes. Further, considering an average of 4933 proteins coded in each genome of the 28 strains, the core compartment (2024 clusters) represent approximately 42% of the total genome (Supplementary Table S3). On the other hand, the accessory genome size ranged from 180 to 2736 clusters among the strains (Figure 3b). Remarkably, the accessory genome of *Pseudomonas* sp. GC01 is composed of 271 clusters, 24 of them exclusively found on this strain (22 hypothetical proteins and two possible related to stress response: the SOS response associated protein UmuD and a FAD-dependent oxidoreductase). Moreover, our data show that there is a significant fraction of the *Pseudomonas* sp. GC01 genome that we cannot discard as the possible origin of the particular capacities displayed by the strain. Therefore, with the improvement of bioinformatics tools and database enrichment, these mysteries will be cleared up over time [56].

### 3.3. Comparative Overview of Metal-Resistance Genes on Pseudomonas Strains

Cadmium is a heavy metal with no cellular role and highly toxic for most organisms. Cadmium toxicity is associated with oxidative stress and damage to different cellular biomolecules, such as lipids, proteins, and nucleic acids [57,58,59,60,61]. *Pseudomonas* sp. GC01 is a Cd-resistant (1.8 mM CdCl_2_) strain that biosynthesizes CdS QDs when exposed to this metal [6,8]. However, the genes involved in Cd^+2^ tolerance/response and their implication in the biosynthesis of nanoparticles are still unknown. Based on these, we performed a bioinformatic search of metal resistance genes focusing on the Cd-resistance markers in all 28 *Pseudomonas* genomes, using the BacMet database [44] and the UniProt entries for a more specific search. 

**Figure 4:**
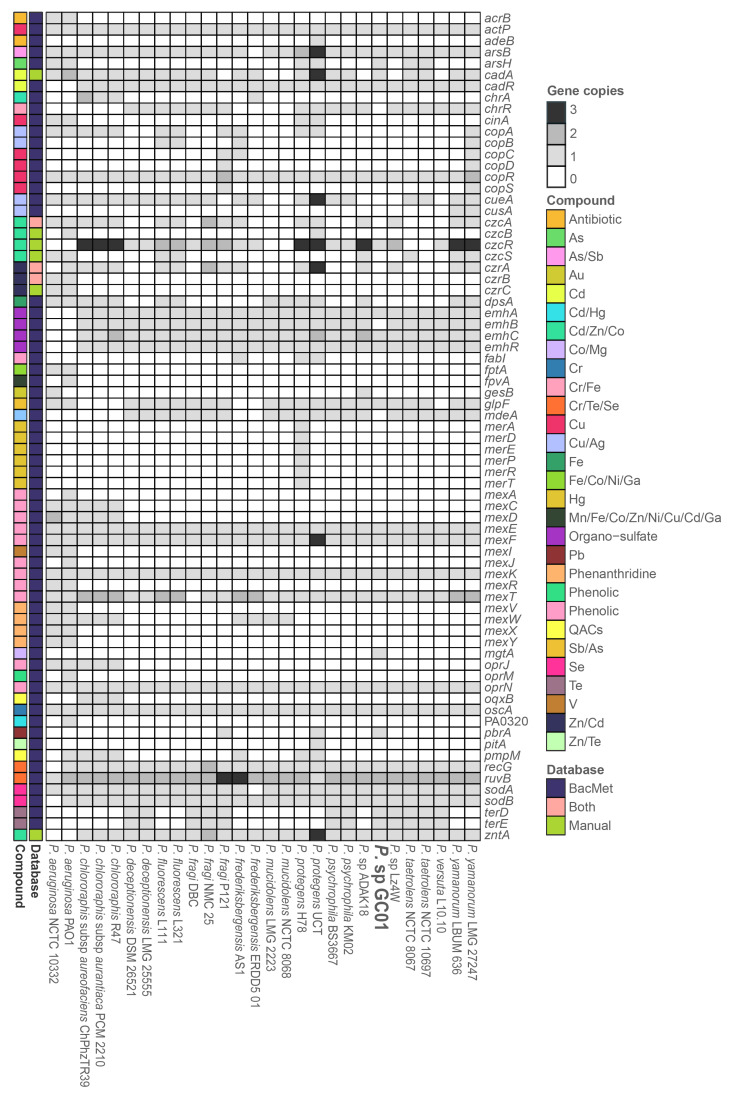
Heatmap of the metal-resistance genes present on the 28 *Pseudomonas* genomes. The scale shows the copy number of each gene in the corresponding genome, the metal(loid)/compound associated with the gene, and the database used.

*Pseudomonas* genomes revealed 72 genes involved in the resistance to multidrug, oxidative stress, and metal(loid), among others (Figure 4). Forty-seven genes are involved in the uptake, tolerance, or detoxification of metal(loid)s, such as arsenic (As), antimony (Sb), zinc (Zn), iron (Fe), copper (Cu), magnesium (Mg), manganese (Mn), chromium (Cr), tellurium (Te), selenium (Se), silver (Ag), cobalt (Co), lead (Pb), mercury (Hg), nickel (Ni), and Cd (Figure 4). Four of these genes, *actP*, *copR*, *oscA*, and *ruvB*, were present in all *Pseudomonas* strains, while other five genes, *arsB*, *cadR*, *recG*, *sodA*, and *sodB*, were absent only in one or two strains of the dataset (Figure 4). *P. aeruginosa* PAO1 (*fpvA* and *PA0320*), *P. protegens* UCT (*merA*, *merD*, *merE*, *merP*, *merR*, *merT* and *pitA*), and *Pseudomonas* sp. GC01 (*mgtA*) presented exclusive metal(loid) resistance genes (Figure 4), according to the BacMet database. 

**Figure 5:**
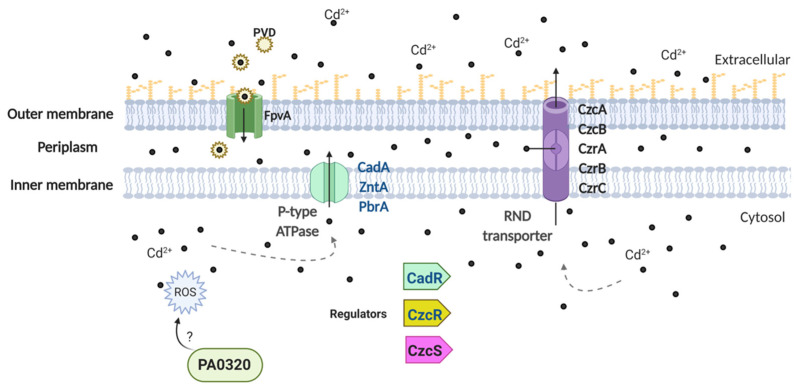
Schematic representation of the cadmium-resistance mechanisms based on the *Pseudomonas* genomes results. Protein names in blue are those found in the genome of *Pseudomonas* sp. GC01.

In general, the analysis of the 28 *Pseudomonas* strains indicated the presence of at least one gene related to cadmium in each genome, and a total of thirteen genes when all genomes were considered (*cadA*, *zntA*, *pbrA*, *cadR*, *czcR*, *czcS*, *czcA*, *czcB*, *czrA*, *czrB*, *czrC*, *fpvA*, and *PA0320*; Figure 4 and Figure 5). Most of these genes correspond to classic genomic determinants involved in cadmium response [60,62] that code for bivalent cation transport pumps and regulatory systems (Figure 5) [61,63,64]. 

Five genes associated with Cd response were determined in the genome of *Pseudomonas* sp. GC01 (Figure 4 and Figure 5). Three of these code for the PIB2-type ATPases (TC 3.A.3) *cadA* (Cd^2+^ and Zn^2+^), *zntA* (Zn^2+^, Cd^2+^, and Pb^2+^), and *pbrA* (Pb^2+^, Zn^2+^, and Cd^2+^), which are primarily involved in cadmium, zinc, and lead transport from the cytoplasm to the periplasm [61,64,65,66,67] (Figure 5). The other two genes, *cadR* and *czcR*, code for the regulatory elements involved in metal resistance (Figure 5). *cadR* encodes a cadmium-induced transcriptional regulatory protein involved in Cd^2+^ resistance in several bacteria, including *Pseudomonas* strains [68,69,70]. While, *czcR* has been described as a DNA-binding heavy metal response regulator, part of the *czcRS* two-component system, involved in Cd^2+^, Zn^2+^, and Co^2+^ resistance [69,71,72,73,74]. The five genes present in *Pseudomonas* sp. GC01 were found in most *Pseudomonas* strains; *cadA* and *czcR* in 22 strains, *zntA* and *cadR* in 26 strains, and *pbrA* only in 3 strains (Figure 4). 

Regarding the Cd^2+^ resistance genes *czcA*, *czcB*, *czcS*, *czrA*, *czrB*, *czrC*, *fpvA*, and *PA0320*, none of them was found in the genome of *Pseudomonas* sp. GC01 (Figure 4). *czcA* and *czcB* (Cd^2+^, Zn^2+^, and Co^2+^), as well as *czrA*, *czrB*, and *czrC* (Cd^2+^, and Zn^2+^), are members of the RND family (resistance–nodulation–cell division, TC_2.A.6.3) of heavy metal efflux, that are involved in the export of Cd^+2^ from the periplasm to the extracellular space [60,75,76,77,78,79]. The czc family is one of the best characterized RND efflux outer membrane proteins involved in Cd^2+^ resistance present in many Gram-negative bacteria, including *Pseudomonas* [58,71,72,73,80]. However, *czcA* and *czcB* were absent in 12 and 26 *Pseudomonas* genomes, respectively, including *Pseudomonas* sp. GC01 (Figure 4). The genes involved in the *czr* efflux system were absent in 13 (*czrA*) and 26 (*czrB*, and *czrC*) *Pseudomonas* strains (Figure 4). Additionally, the regulatory gene *czcS* (part of the two-component-regulatory systems *czcRS*) was present in 8 *Pseudomonas* strains (Figure 4). This gene encodes a heavy metal sensor histidine kinase involved in Cd^2+^, Zn^2+^, and Co^2+^ homeostasis in bacteria [67,72]. On the other hand, the gene *PA0320* has been associated with Cd^2+^ resistance by favoring the tolerance to reactive oxygen species (ROS; [81]). The *fpvA* gene (Fe^3+^, Mn^2+^, Co^2+^, Zn^2+^, Ni^2+^, Cu^2+^, and Cd^2+^) codes for an outer membrane transporter and receptor of the siderophore pyoverdine (PVD), involved in iron uptake with a broad specificity for PVD–metal complexes in *P. aeruginosa* [82,83]. These last two genes were only found in *P. aeruginosa* PAO1 (Figure 4).

According to the results obtained, the absence of *czcA*, *czcB*, *czrA*, *czrB*, and *czrC* in *Pseudomonas* sp. GC01 denies the possibility of their participation in the Cd^2+^ transport process required for the extracellular biosynthesis of CdS. However, *Pseudomonas* sp. GC01 contains other genes involved in the cadmium response (Figure 4) that could participate in the biosynthesis of CdS QDs. *Pseudomonas* sp. GC01 *cadA*, *zntA*, and *pbrA* genes are candidates for Cd^2+^ efflux, favoring the extracellular interaction of the metal ions with sulfur-containing molecules, such as H_2_S or MeSH, to form the CdS nanoparticles. These genes represent three potential targets probably involved in CdS nanoparticle formation in *Pseudomonas* sp. GC01. However, the most novel characteristic of CdS biosynthesis in this bacterium is their capacity to synthesize nanoparticles in the presence of different sulfur sources, particularly Met. 

### 3.4. Comparative Analysis of Genes Involved in Sulfur Metabolism

**Figure 6:**
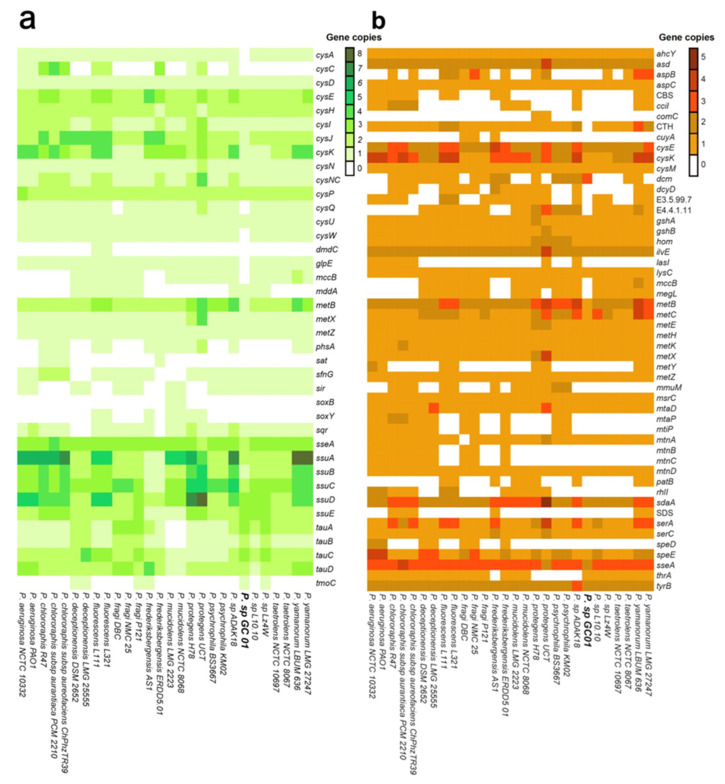
Heatmap of the sulfur metabolic genes present in the 28 *Pseudomonas* genomes analyzed. (**a**) Sulfur metabolism genes. (**b**) Cysteine and methionine metabolism genes. The heat scale shows the copy number of each gene in the corresponding genome.

Since the *Pseudomonas* sp. GC01 can biosynthesize CdS QDs from several sulfur sources, and sulfur is a vital element in the formation of these nanoparticles, we searched for genes involved in sulfur metabolic pathways. To carry out this, we use data available in the KEGG database related to sulfur metabolism (map00920) and cysteine/methionine metabolism (ko00270). A set of 91 genes were found in the 28 *Pseudomonas* genomes, 39 belonging to sulfur metabolism, 52 to cysteine/methionine metabolism, and seven shared in both metabolisms (Figure 6). The genome sequences revealed the presence of numerous common genes encoding proteins related to sulfur transport, sulfate/sulfur assimilation, Cys and Met synthesis/degradation, and VSCs catabolic pathways, among others. These results are consistent with the ability of bacteria of the *Pseudomonas* genus to use a wide variety of organic and inorganic sulfur sources to grow [84,85]. 

Sulfur is an essential element required for cell growth in all bacteria [86]. It is also a necessary component in CdS nanoparticle biosynthesis [6,24,26,87]. The *Pseudomonas* sp. GC01 strain can use several sulfur sources, such as sulfate, sulfite, thiosulfate, Cys, and Met, to grow and biosynthesize CdS QDs [8]. However, the extracellular biosynthesis mechanism of the CdS nanoparticles has been linked to the ability of this strain to release H_2_S and MeSH in the presence of Cys and Met, respectively [8]. 

**Figure 7:**
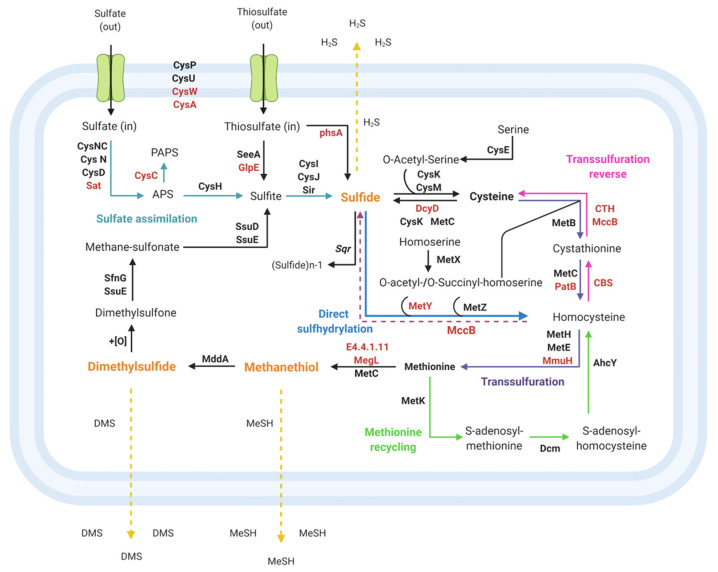
Sulfur metabolic pathways present in the genome of *Pseudomonas* sp. GC01. The schematic representation of protein identified in the genome of *Pseudomonas* sp. GC01 involved in sulfur assimilation, cysteine and methionine synthesis, and volatile sulfur compounds catabolic pathways. Protein names in red were not found in this strain but present in other *Pseudomonas* strains.

Sulfate assimilation in bacteria proceeds by a sequence of similar reactions involving the uptake and activation of sulfate, followed by a stepwise reduction to sulfide [83,88]. The *Pseudomonas* genome sequences revealed that sulfate assimilation begins with the active uptake of sulfate by the ABC-type transport for sulfate/thiosulfate (encoded by *cysA* (EC: 3.6.3.25), *cysP*, *cysU*, and *cysW* in all strains, and by *cysP* and *cysU* in *Pseudomonas* sp. GC01) (Figure 7). Subsequently, sulfate is transformed to adenosine-5′-phosphosulphate (APS), catalyzed by ATP sulfurylase (EC: 2.7.7.4), and encoded by *cysN*, *cysD*, and *cysNC* in the *Pseudomonas* strains [85], and by *sat* genes, which were present only in four strains (Figure 6a). Then APS can be reduced to sulfite directly through APS reductase *cysH* (EC: 1.8.4.10, present in all strains), or indirectly via 3′-phosphoadenosine-5′-phosphosulfate (PAPS) that uses APS kinase *cysC* (EC: 2.7.1.25), followed by PAPS reductase (*cysH*, EC: 1.8.4.8, found in 11 *Pseudomonas* strains) (Figure 6a). The obtained sulfite is then reduced to generate sulfide by sulfite reductase encoded by *cysI*, *cysJ* (EC: 1.8.1.2), and *sir* (EC: 1.8.7.1), found in 19 strains, including *Pseudomonas* sp. GC01), before being assimilated into organic material (Figure 6a and Figure 7) [85,89,90].

Regarding the use of sulfite and thiosulfate as inorganic sulfur sources by bacteria, sulfite can enter the sulfate assimilation pathway, where sulfite is reduced to sulfide by the enzyme sulfite reductase *cysI*, *cysJ*, and *sir* [89]. Thiosulfate can be incorporated through ABC-type transporters (sulfate/thiosulfate, described above) and reduced to sulfite by thiosulfate sulfurtransferase (EC: 2.8.1.1; [91]) encoded by *seeA* and *glpE* (the latter absent only in *Pseudomonas* sp. GC01) before being assimilated as sulfide via the sulfate assimilation pathway (Figure 6a and Figure 7). Additionally, 14 strains of *Pseudomonas* (not including *Pseudomonas* sp. GC01) contain in their genomes the thiosulfate reductase enzyme (*phsA*, EC: 1.8.5.5) that catalyzes the reduction of thiosulfate to sulfide (Figure 6a and Figure 7) [92,93]. The sulfide generated by the different inorganic sulfur sources is incorporated into cells as cysteine by the action of the enzymes cysteine synthase *cysk* and *cysM* (EC: 2.5.1.47 and EC: 2.5.1.144 [94]) that catalyzes the addition of sulfide into O-acetyl-serine (Figure 6a,b and Figure 7). Cys then works as the sulfur group donor, either directly or indirectly, to synthesize all other sulfur-bearing molecules in the cell, such as thiamine, glutathione, coenzyme A, and Met [90,95]. 

Some bacteria belonging to the *Pseudomonas* genus can assimilate sulfur from reduced sulfur molecules, such as the amino acids Cys and Met [84]. Generally, this metabolism is associated with internal recycling processes developed by bacteria to maximize the available nutrients [96]. Cys as a sole sulfur source can be assimilated directly via the transsulfuration pathway that converts Cys to Met (Figure 7) [84,97]. This pathway was observed in all *Pseudomonas* genomes analyzed and produces homocysteine by the action of cystathionine γ-synthetase (*metB*, EC: 2.5.1.48) and cystathionine β-lyase (EC: 4.4.1.13) encoded by *metC* and *patB* (in 9 strains; Figure 6b) [98,99,100]. Subsequently, homocysteine is methylated to produce Met by methionine synthases, encoded by *metE* or *metH* (EC: 2.1.1.14 or EC: 2.1.1.13 [101]) present in all strains, and by homocysteine S-methyltransferase (*mmuH*, EC: 2.1.1.13 [102]), determined in 12 *Pseudomonas* strains (Figure 6b and Figure 7). 

On the other hand, Cys also may break down to release sulfide in bacteria through the enzyme cysteine desulfhydrase (EC: 4.4.1.15) [103,104,105,106]. The gene encoding this enzyme (*dcyD*) was found in 16 strains, not including the *P. aeruginosa*, *P. deceptionensis*, *Pseudomonas* sp. Lz4W, *Pseudomonas fragi* P121, and *Pseudomonas* sp. GC01 strains, among others (Figure 6b). Low H_2_S production in the presence of Cys and other sulfur sources had been reported in *Pseudomonas* sp. GC01 by Gallardo-Benavente et al. (2019) [8]. This report is concordant with the absence of *dcyD* in this strain. The presence of the enzyme sulfide:quinine oxidoreductase, encoded by *sqr* (EC: 1.8.5.4, present in 15 strains; Figure 6a), whose function is to oxidize sulfide to polysulfide or sulfite and thiosulfate in heterotrophic bacteria such as *P. aeruginosa* PAO1 [107], diminishes the sulfide released by bacteria. Therefore, the *dcyD* absence in *Pseudomonas* sp. GC01 suggests that sulfide production from Cys may be catalyzed by enzymes with lower cysteine desulfhydrase activity present in their genome, such as cysteine synthases (*cysK*) and cystathionine β-lyase (*metC*) (Figure 7) [103,104].

The sulfide produced by the sulfate assimilation pathway or Cys degradation can be used by *Pseudomonas* strains to yield Met through transsulfuration (described above) and direct sulfhydrylation pathways. The direct sulfhydrylation pathway has been described as the main Met synthesis pathway in the *Pseudomonas* genus [85], and the critical gene (*metZ*) was present in all genomes analyzed (Figure 6b and Figure 7). Before Met formation, this pathway involves the direct formation of homocysteine catalyzed by the enzyme O-succinylhomoserine sulfhydrylase (*metZ*, EC:2.5.1.-), using O-succinyl-homoserine as substrate (Figure 6b), or the synthesis of homocysteine using O-acetyl-homoserine and sulfide (present in 8 strains) catalyzed by the O-acetyl-L-homoserine sulfhydrolase (*metY*, EC: 2.5.1.49) [84,85,97,101,108,109,110]. 

Regarding the metabolic pathways associated with Met as a sole sulfur source for bacterial growth, the *Pseudomonas* genomes showed two metabolic pathways that allow conversion of this amino acid to Cys for cell growth. In the first pathway, Met may be desulfurized to produce sulfite entering in the synthetic pathway of Cys through sulfate assimilation [84]. The formation of MeSH from Met degradation catalyzed by enzyme methionine γ-lyase (EC: 4.4.1.11) has been reported in *Pseudomonas* [47,111,112]. The genes coding for this enzyme, *megL* and *EC4.4.1.11*, were found in 19 and 20 of the *Pseudomonas* genomes analyzed, respectively (Figure 6b). Despite the ability of *Pseudomonas* sp. GC01 to produce high concentrations of MeSH from Met, these genes were absent in their genome [8]. This result suggests that MeSH production in *Pseudomonas* sp. GC01 is catalyzed by an enzyme different from methionine γ-lyase. In this context, the enzyme cystathionine β-lyase (*metC*) has been described in some bacteria with the ability to produce MeSH from Met [113,114,115,116] and is presented as the principal candidate to carry out this function in the *Pseudomonas* sp. GC01 (Figure 7). Once MeSH is formed, this sulfur volatile is methylated by the enzyme methanethiol S-methyltransferase (*mddA*, EC: 2.1.1.334) to produce dimethylsulfide (DMS) [47], and then DMS is oxidized to dimethylsulfone [117]. Dimethylsulfone is converted to methane-sulfonate and sulfite by the action of the enzymes dimethylsulfone monooxygenase (*sfnG*, EC: 1.14.14.35), FMN reductase (*ssuE*, EC 1.5.1.38), and alkanesulfonate monooxygenase (*ssuD*, EC: 1.14.14.5) [117]. Finally, the sulfite produced can enter the sulfate assimilation pathway for Cys biosynthesis (Figure 7) [84,117]. Two key members of Met desulfurization to sulfite are the *mddA* and *sfnG* genes, which were absent in 17 and 5 *Pseudomonas* strains analyzed, respectively (Figure 6a). Therefore, this result showed the presence of this pathway in nine bacterial strains, including *Pseudomonas* sp. GC01 (Figure 6a and Figure 7). 

The second pathway that converts Met to Cys in bacteria involves methionine recycling and homocysteine conversion to Cys by reverse transsulfuration or sulfide formation (Figure 7) [84,85,118]. In general, Met is used by S-adenosylmethionine synthase (*metK*, EC: 2.5.1.16), present in all genomes, for the synthesis of the universal methyl donor S-adenosyl-methionine (SAM) [85,97]. Subsequently, SAM is regenerated or recycled to Met via the formation of homocysteine by the action of methyltransferases (encoded by *dmc* (EC: 2.1.1.37, cytosine-specific methyltransferases) in 18 strains) and homocysteine adenosylhomocysteine *ahcY* (EC: 3.3.1.1) (Figure 6b and Figure 7). Then, Cys is formed by the reverse transsulfuration pathway via cystathionine by the enzymes cystathionine β-synthase (*CBS*, EC: 4.2.1.22) and cystathionine γ-lyase (*CTH* or *mccB*, EC: 4.4.1.1) in 12 strains (Figure 6b and Figure 7). While, in 17 *Pseudomonas* strains, homocysteine desulfhydrase *mccB* (EC: 4.4.1.2) produces sulfide from homocysteine, which enters the Cys biosynthesis pathway via the sulfate assimilation route (Figure 7). *Pseudomonas* sp. GC01 lacks *CBS*, *CTH*, and *mccB* genes, suggesting that Met is used as a sulfur source in this bacterium by the Met desulfurization pathway via dimethylsulfone. However, we cannot discard that other still unknown genes or pathways could be involved in this process. 

In general, no significant discrepancies were observed between the 28 *Pseudomonas* genomes analyzed. From a total of 84 genes observed, 33 genes were absent in some of the bacterial strains (Figure 6), probably due to the strains’ genomic diversity (Figure 2 and Figure 3). Nevertheless, sulfur is an essential element for bacterial growth. Therefore, the sulfur metabolism pathways are remarkably similar between different organisms [85,86]. Bacterial assimilation of sulfur into organic molecules is varied, and the sulfur metabolic pathways depend on the sulfur source used and the genomic potential of each bacteria. The genes involved in sulfate assimilation pathways, transsulfuration, direct sulfhydrylation, methionine salvage, reverse transsulfuration, VSCs catabolic pathways, among others described in this work, were found in the *Pseudomonas* strains analyzed (Figure 7).

Extracellular biosynthesis of CdS QDs in bacteria has been mainly associated with H_2_S [24,87] and recently has been linked with MeSH production in *Pseudomonas* sp. GC01 [8]. When Cys is used as the sulfur source, the mechanism of CdS nanoparticles involves H_2_S generation (as sulfide source) mediated by cysteine desulfhydrase (*dcyD*) or cystathionine γ-lyase (*CTH* or *mccB*) [26,87,119]. Interestingly, *Pseudomonas* sp. GC01 biosynthesizes CdS nanoparticles from Cys, despite lacking these genes. Therefore, it is believed that *cysK* (cysteine synthases) and *metC* (cystathionine β-lyase) would participate in H_2_S production in this strain. 

Regarding the CdS QD biosynthesis associated with the production of MeSH in bacteria, Gallardo-Benavente et al. (2019) proposed a relationship between CdS nanoparticle biosynthesis and MeSH production in *Pseudomonas* sp. GC01, which was also linked to methionine γ-lyase activity in *P. deceptionensis* M^1T^ [8]. However, the *megL* and *EC4.4.1.11* genes that encode this enzyme are absent in the *Pseudomonas* sp. GC01 genome. This is puzzling, particularly considering the high MeSH production and the ability to produce CdS QDs in the presence of Met, as previously reported in this strain [8]. Accordingly, once again, *metC* appears as the primary candidate to carry out this function due to their ability to produce MeSH from Met in bacteria [113,114,115,116]. 

## 4. Conclusions

**Figure 8:**
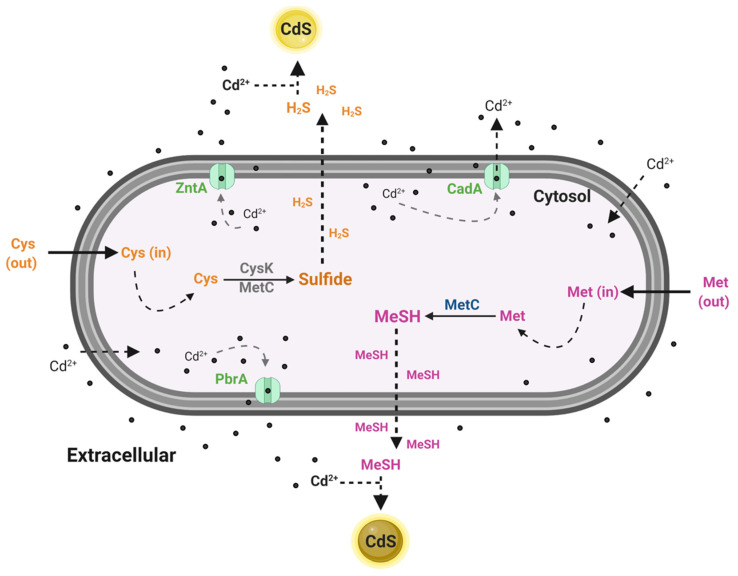
Schematic representation of the CdS QDs biosynthesis by *Pseudomonas* sp. GC01. The figure shows the proteins present in the *Pseudomonas* sp. GC01 genome involved in the biosynthesis of CdS nanoparticles when Cys (CysK and MetC) or Met (MetC) was used as the sulfur source as well as the Cd^2+^ efflux pumps CadA, ZntA, and PbrA.

Our findings confirm the presence of three Cd efflux P-type ATPases transporters (*cadA*, *zntA*, and *pbrA*) in the genome of *Pseudomonas* sp. GC01. Identifying these Cd^2+^ transport genes provides evidence about the detoxification mechanisms of cadmium in this strain, which could contribute to the extracellular biosynthesis of CdS QDs (Figure 8). 

The absence of *megL* and *EC4.4.1.11* in the *Pseudomonas* sp. GC01 genome discarded a role for the enzyme methionine γ-lyase in the MeSH-dependent biosynthesis of QDs CdS. However, the *metC* gene (coding for cystathionine β-lyase) involved in the transsulfuration pathway is the primary candidate to produce MeSH from Met during the extracellular biosynthesis of CdS QDs in *Pseudomonas* sp. GC01 (Figure 8). In addition, H_2_S generation from Cys during the extracellular biosynthesis of CdS nanoparticles is most probably linked to *cysK* (cysteine synthases) and *metC* in *Pseudomonas* sp. GC01, since this strain lacks the gene *dcyD* coding for cysteine desulfhydrase (Figure 8).

Altogether, the results presented in this study constitute valuable information regarding the potential molecular mechanism involved in the bacterial biosynthesis of CdS QDs based on H_2_S and MeSH generation, two processes scarcely known to date. Therefore, this genomic study constitutes the first report about the genes potentially involved in CdS QDs bioproduction in *Pseudomonas* sp. strains and the first molecular approach to a bacterial mechanism of Cd-resistance and MeSH production in the Antarctic strain *Pseudomonas* sp. GC01.

## Data Availability

The complete genome sequence of *Pseudomonas* sp. strain GC01 has been deposited in GenBank under the accession number JABEMH000000000.1 (BioProject: PRJNA629082).

## Supplementary Materials

The following are available online at https://www.mdpi.com/2073-4425/12/2/187/s1, Table S1: Accession numbers and data for the selected 28 *Pseudomonas* strains genomes, Table S2: Cadmium-resistance Genes Database described in the literature to *Pseudomonas*, Table S3: Genes Classification for each strain in the pangenome compartments.
