# Supplementary material for: Genomics Insights into Pseudomonas sp. CG01: An Antarctic Cadmium-Resistant Strain Capable of Biosynthesizing CdS Nanoparticles Using Methionine as S-Source
Source: Genes (Basel). 2021 Jan 27;12(2):187. doi: 10.3390/genes12020187 (PMC7912247; doi:10.3390/genes12020187)
Supplement: Supplementary file 1 [file genes-12-00187-s001.pdf]

Supplementary Materials

Supplementary Table S1. Accession numbers and data for the selected 28 *Pseudomonas* strains genomes.

Species	Strain	BioSample	BioProject	Sample	Geographic location
Pseudomonas sp	Lz4W	SAMN02469436	PRJNA170013	Soil	Schirmacher Oasis, Antarctica
Pseudomonas sp	L10.10	SAMN04076495	PRJNA295629	Soil	Antarctica
Pseudomonas sp	ADAK18	SAMN14692983	PRJNA627971	boreal fostest	Alaska, USA
Pseudomonas psychrophila	KM02	PRJNA509367	SAMN14133006	Food spoilage microflora	Poland
Pseudomonas psychrophila	BS3667	SAMN04490201	PRJEB16505	missing	missing
Pseudomonas taetrolens	NCTC10697	SAMEA3711430	PRJEB6403	not available	not available
Pseudomonas taetrolens	NCTC8067	SAMEA26390668	PRJEB6403	not available	not available
Pseudomonas fragi	P121	SAMN04371283	PRJNA307076	Sediment	Artic
Pseudomonas fragi	NMC25	SAMN06628701	PRJNA380155	meat	China
Pseudomonas fragi	DBC	SAMN07187748	PRJNA388845	PAH contaminated soil	India
Pseudomonas chlororaphis	R47	SAMN06241861	PRJNA355625	rhizosphere soil	Switzerland
Pseudomonas chlororaphis subsp. aureofaciens	ChPhzTR39	SAMN08359181	PRJNA433211	tomato rhizosphere	France: Provence-Alpes-Cote d'Azur, Chateaurenard
Pseudomonas chlororaphis subsp. aurantiaca	PCM 2210	SAMN08359189	PRJNA433211	sugar-beetroot rhizosphere	Poland
Pseudomonas protegens	H78	SAMN04240923	PRJNA301182	soil	China
Pseudomonas protegens	UCT	SAMN05964115	PRJNA299395	contaminated site	Czech Republic
Pseudomonas fluorescens	L321	SAMN04992557	PRJNA320923	temperate forest	Ireland
Pseudomonas fluorescens	L111	SAMN04992704	PRJNA320923	temperate forest	Ireland
Pseudomonas frederiksbergensis	ERDD5:01	SAMN05947123	PRJNA350793	glacier stream	India
Pseudomonas frederiksbergensis	AS1	SAMN06102480	PRJNA343270	arsenic-contaminated soil	South Korea
Pseudomonas mucidolens	LMG2223	SAMN05216202	PRJEB16499	missing	missing
Pseudomonas mucidolens	NCTC8068	SAMEA4040591	PRJEB6403	missing	missing
Pseudomonas yamanorum	LMG 27247	SAMN05216237	PRJEB16453	missing	missing
Pseudomonas yamanorum	LBUM636	SAMN03981702	PRJNA292571	field soil	Canada
Pseudomonas sp.	GC01	SAMN14766589	PRJNA629082	soil	Antarctica: Deception Island, South Shetland Archipelago
Pseudomonas aeruginosa	NCTC10332	SAMEA2479570	PRJEB6403	not available	Czech Republic
Pseudomonas aeruginosa	PAO1	SAMN02603714	PRJNA331	not available	missing
Pseudomonas deceptionensis	LMG 25555	SAMN04489800	PRJEB16503	missing	missing
Pseudomonas deceptionensis	DSM 26521	SAMN03328792	PRJNA274345	marine sediment	Antartica

Supplementary Table S2. Cadmium-resistance Genes Database described in the literature to *Pseudomonas*.

Gene	Family	Substrates	Organism	Reference
cadA			*P. putida* 06909	[1]
	P-type ATPases	Cd^{2+}, Zn^{2+}	*P. sp. MPC6*	[2]
cadA2R			*P. putida* CD2	[3]
			P. aeruginosa	[4]
czcA	Cation diffusion facilitator	Cd^{2+}, Zn^{2+}, Co^{2+}	*P.* sp. MPC6	[2]
			P. sp. P11	[5]
	Membrane fusion protein	Cd^{2+}, Zn^{2+}, Co^{2+}	*P. aeruginosa*	[4]
czcB			*P.* KT2440	[2]
	Cation diffusion facilitator		*P. aeruginosa*	[4]
czcC		Cd^{2+}, Zn^{2+}, Co^{2+}	*P. putida* KT2440	[2]
			P. sp. MPC6	[2]
czcR	Regulator	Cd^{2+}, Zn^{2+}, Co^{2+}	*P. aeruginosa*	[6]
czcS			*P. aeruginosa* PAO1	[7]
			P. putida X4	[8]
CzrA				
CzrB	RND Efflux	Cd^{2+}, Zn^{2+}	*P. aeruginosa* CMG103	[9]
CzrC				
ZntA	P-type ATPases	Pb^{2+}, Cd^{2+}, Zn^{2+}	*Pseudomonas fluorescens* strain ATCC 13525	[10]

RND: Resistance-nodulation-division.

Supplementary Table S3. Genes Classification for each strain in the pangenome compartments.

Strain	Total Proteins	Accessory	Disposable	Core
P. aeruginosa NCTC10332	5704	2736 (48.0%)	944 (16.5%)	2024 (35.5%)
P. aeruginosa PAO1	5694	2720 (47.8%)	950 (16.7%)	2024 (35.5%)
P. chlororaphis R47	6042	1038 (17.2%)	2980 (49.3%)	2024 (33.5%)
P. chlororaphis subsp. aurantiaca PCM2210	5631	726 (12.9%)	2881 (51.2%)	2024 (35.9%)
P. chlororaphis subsp. aureofaciens ChPhzTR39	5911	979 (16.6%)	2908 (49.2%)	2024 (34.2%)
P. deceptionensis DSM26521	4560	386 (8.5%)	2150 (47.1%)	2024 (44.4%)
P. deceptionensis LMG25555	4561	387 (8.5%)	2150 (47.1%)	2024 (44.4%)
P. fluorescens L111	5820	1042 (17.9%)	2754 (47.3%)	2024 (34.8%)
P. fluorescens L321	5844	1042 (17.8%)	2778 (47.5%)	2024 (34.6%)
P. fragi DBC	3908	180 (4.6%)	1704 (43.6%)	2024 (51.8%)
P. fragi NMC25	4182	247 (5.9%)	1911 (45.7%)	2024 (48.4%)
P. fragi P121	4316	359 (8.3%)	1933 (44.8%)	2024 (46.9%)
P. frederiksbergensis AS1	4495	285 (6.3%)	2186 (48.6%)	2024 (45.0%)
P. frederiksbergensis ERDD5:01	4197	336 (8.0%)	1837 (43.8%)	2024 (48.2%)
P. mucidolens LMG2223	5242	981 (18.7%)	2237 (42.7%)	2024 (38.6%)
P. mucidolens NCTC8068	5241	980 (18.7%)	2237 (42.7%)	2024 (38.6%)
P. protegens H78	5494	790 (14.4%)	2680 (48.8%)	2024 (36.8%)
P. protegens UCT	4379	804 (18.4%)	1551 (35.4%)	2024 (46.2%)
P. psychrophila BS3667	4710	529 (11.2%)	2157 (45.8%)	2024 (43.0%)
P. psychrophila KM02	4717	532 (11.3%)	2161 (45.8%)	2024 (42.9%)
P. sp. ADAK18	5031	334 (6.6%)	2673 (53.1%)	2024 (40.2%)
P. sp. GC01	4875	271 (5.6%)	2580 (52.9%)	2024 (41.5%)
P. sp. L10.10	4132	167 (4.0%)	1941 (47.0%)	2024 (49.0%)
P. sp. Lz4W	4298	277 (6.4%)	1997 (46.5%)	2024 (47.1%)
P. taetrolens NCTC10697	4305	536 (12.5%)	1745 (40.5%)	2024 (47.0%)
P. taetrolens NCTC8067	4368	562 (12.9%)	1782 (40.8%)	2024 (46.3%)
P. yamanorum LBUM636	5907	879 (14.9%)	3004 (50.9%)	2024 (34.3%)
P. yamanorum LMG27247	5929	893 (15.1%)	3012 (50.8%)	2024 (34.1%)

Reference

1. Lee, S.W.; Glickmann, E.; Cooksey, D.A. Chromosomal locus for cadmium resistance in *Pseudomonas putida* consisting of a cadmium-transporting ATPase and a MerR family response regulator. *Appl. Environ. Microbiol*. **2001**, *67*, 1437-1444.
DOI: 10.1128/AEM.67.4.1437-1444.2001

2. Orellana-Saez, M.; Pacheco, N.; Costa, J.I.; Mendez, K.N.; Miossec, M.J.; Meneses, C.; Castro-Nallar, E.; Marcoleta, A.E.; Poblete-Castro, I. In-depth genomic and phenotypic characterization of the Antarctic psychrotolerant strain *Pseudomonas* sp. MPC6 reveals unique metabolic features, plasticity, and biotechnological potential. *Front. Microbiol*. **2019**, *10*, 1154.

3. Hu, N.; Zhao, B. Key genes involved in heavy-metal resistance in *Pseudomonas putida* CD2. *FEMS Microbiol. Lett*. **2007**, *267*, 17-22.
DOI:https://doi.org/10.1111/j.1574-6968.2006.00505.x

4. Joonu, J.; Averal, H.I. Heavy metal resistant CZC genes identification in *Bacillus cereus*, *Enterobacter asburiae* and *Pseudomonas aeruginosa* isolated from BHEL industry, Tamilnadu. *J. Microbiol. Biotechnol*. **2016**, **5**, 27-31.
DOI: https://doi.org/10.3389/fmicb.2019.01154

5. Yan, Z.; Li, M.; Wang, J.; Pan, J. Genome Analysis Revealing the Potential Mechanisms for the Heavy Metal Resistance of *Pseudomonas* sp. P11, Isolated from Industrial Wastewater Sediment. *Curr. Microbiol*. **2019**, *76*, 1361-1368.
DOI: https://doi.org/10.1007/s00284-019-01728-2

6. Wang, D.; Chen, W.; Huang, S.; He, Y.; Liu, X.; Hu, Q.; Wei, T.; Shang, H.; Gan,J .; Chen, H. Structural basis of Zn (II) induced metal detoxification and antibiotic resistance by histidine kinase CzcS in *Pseudomonas aeruginosa*. *PLoS Pathog*. **2017**, *13*, e1006533.
DOI:https://doi.org/10.1371/journal.ppat.1006533

7. Perron, K.; Caille, O.; Rossier, C.; Van Delden, C.; Dumas, J. L.; Köhler, T. CzcR-CzcS, a two-component system involved in heavy metal and carbapenem resistance in *Pseudomonas aeruginosa*. *J. Biol. Chem*. **2004**, *279*, 8761-8768.
DOI:doi: 10.1074/jbc.M312080200

8. Liu, P.; Chen, X.;Huang, Q.; Chen, W. The role of CzcRS two-component systems in the heavy metal resistance of *Pseudomonas putida* X4. Int. J. Mol. Sci. 2015, 16, 17005-17017.
DOI: https://doi.org/10.3390/ijms160817005

9. Hassan, M.; van der Lelie, D.; Springael, D.; Römling, U.; Ahmed, N.; Mergeay, M. Identification of a gene cluster, czr, involved in cadmium and zinc resistance in *Pseudomonas aeruginosa*. *Gene* **1999**, *238*, 417-425.
DOI:https://doi.org/10.1016/S0378-1119(99)00349-2

10. Rossbach, S.; Wilson, T.L.; Kukuk, M.L.; Carty, H.A. Elevated zinc induces siderophore biosynthesis genes and a zntA-like gene in *Pseudomonas fluorescens*. *FEMS Microbiol. Lett*. **2000**, *191*, 61-70.
DOI:https://doi.org/10.1111/j.1574-6968.2000.tb09320.x
